# Success of the Eight-Hour Day

**Published:** 1921-02-19

**Authors:** 


### THE MATRONS' AND SISTERS' DEPARTMENT.

### SUCCESS OF THE EIGHT-HOUR DAY.

No item in hospital expenditure is so unremunerative and unjustifiable as that spent on sick nurses. Few people realise what the normal sickness rate in a large hospital means. The daily average of those off duty on this account varies very largely. In many hospitals no attempt is made to estimate it. In others careful statistics are kept of the number of nurses away on sick leave and in the sick-room, and except under most favourable conditions the percentage is rarely under five. Under modern conditions allowance has to be made for these five per cent. With a nursing staff of 300 it means that fifteen extra nurses have to be kept to fill the place of those who happen to be incapacitated, and since taking it all round every nurse certainly costs the hospital not less than £150 a year we have a total of £2,250 a year at the lowest estimate expended on this item alone. The cost of the sick nurse does not end here. Sick-rooms must be provided, with extra attendance and treatment. In many cases the general health deteriorates in such a way that the services of a valuable nurse or half-trained probationer are lost to the hospital, and the wastage of vitality to the individual thus checked in her career cannot in any degree be estimated. Reckoning the space occupied by the nurses' sick quarters and the expense often incurred by having to engage private nurses to fill the vacancies, it is not extravagant to estimate the cost of sickness among the nursing staff in large establishments at a figure between £5,000 and £10,000 a year.

It is a remarkable fact that at the Charing Cross Hospital, where an approximation to the eight-hour day, with one day's rest in seven, has now been in operation for two years, under the matronship of Miss E. F. Tice, the sickness rate has almost disappeared. The situation of the hospital is not ideal from a health point of view. It lies in a crowded area, and the outlook from the Nurses' Home affords less sunshine and open space than can be approved on strictly hygienic lines. Before the curtailment of duty hours and the institution of the weekly day of rest the sickness rate was higher than in many other hospitals, certainly much over the five per cent. average. The change wrought by providing the nurses with reasonable hours of recreation and lessening the strain of their busy lives has been something like magical.

Miss Tice has not found that her nurses abuse their privileges, amuse themselves too violently or set other interests before those of the hospital. She trusts them and they respond loyally to the spirit of their training-school. Strictly speaking the day is slightly below the eight-hour standard by about half an hour; the hours on duty for the week total fifty. Nurses may sleep away from the hospital on the eve of their day off, and the night nurses have their two days off duty together every twelve days, so that they get a really refreshing interval of rest.

The value to the hospital of securing that all the nurses are fit and enjoy their work is incalculable. And even in money it is clearly better to pay for an extra fifteen or twenty healthy nurses than to house and wait upon the same number of invalids. These facts need to be widely proclaimed. The managers of our hospitals are business men and this is a business proposition. It has been often pointed out that probationers from a health point of view are picked women, selected after a careful medical examination. Once it has been demonstrated that the cause of their crumpling up in training is long hours on duty and lack of a weekly rest, the economy of strengthening their numbers and eliminating the sickness rate is irresistible.

There is another point of view. Probationers are hard to attract at the moment. Candidates if they are wisely advised choose the training-schools which treat them with consideration. At Charing Cross they have again reached the stage when they are inundated with applications. This also makes for economy, for it is possible to spend hundreds of pounds in advertising and get but a poor choice in the end. As a proved business proposition the 48- or 50-hour week with the day's rest once a week must soon become universal.

